# Development of an orally-administrative MELK-targeting inhibitor that suppresses the growth of various types of human cancer

**DOI:** 10.18632/oncotarget.790

**Published:** 2012-12-21

**Authors:** Suyoun Chung, Hanae Suzuki, Takashi Miyamoto, Naofumi Takamatsu, Ayako Tatsuguchi, Koji Ueda, Kyoko Kijima, Yusuke Nakamura, Yo Matsuo

**Affiliations:** ^1^ Department of Medicine and Surgery, The University of Chicago, Chicago, IL, USA; ^2^ OncoTherapy Science, Inc., Kawasaki, Kanagawa, Japan; ^3^ Laboratory for Biomarker Development, RIKEN, Yokohama, Japan; ^4^ Laboratory of Molecular Medicine, Human Genome Center, Institute of Medical Science, The University of Tokyo, Tokyo, Japan

**Keywords:** oncogene, drug discovery, kinase inhibitor, cancer stem cell

## Abstract

We previously reported MELK (maternal embryonic leucine zipper kinase) as a novel therapeutic target for breast cancer. MELK was also reported to be highly upregulated in multiple types of human cancer. It was implied to play indispensable roles in cancer cell survival and indicated its involvement in the maintenance of tumor-initiating cells. We conducted a high-throughput screening of a compound library followed by structure-activity relationship studies, and successfully obtained a highly potent MELK inhibitor OTSSP167 with IC_50_ of 0.41 nM. OTSSP167 inhibited the phosphorylation of PSMA1 (proteasome subunit alpha type 1) and DBNL (drebrin-like), which we identified as novel MELK substrates and are important for stem-cell characteristics and invasiveness. The compound suppressed mammosphere formation of breast cancer cells and exhibited significant tumor growth suppression in xenograft studies using breast, lung, prostate, and pancreas cancer cell lines in mice by both intravenous and oral administration. This MELK inhibitor should be a promising compound possibly to suppress the growth of tumor-initiating cells and be applied for treatment of a wide range of human cancer.

## INTRODUCTION

Breast cancer is the most common malignancy among women worldwide[1]. More than 1.3 million patients are newly diagnosed with breast cancer each year, and over 400,000 patients died of the disease[2]. Treatments acting on molecular targets such as estrogen receptor or HER-2 for breast cancer have successfully improved the mortality rate, but a subset of the patients can still have little benefit with these therapies[3, 4]. Triple-negative breast cancer (TNBC), one of the breast cancer subtypes, develops more frequently in younger women and is known to be more aggressive with poor prognosis[5]. Since TNBC does not expresses either of HER-2, estrogen receptor, or progesterone receptor[6], no effective targeted therapy is presently available[5, 7]. Hence, the development of novel targeted drugs for such patients is urgently awaited.

We identified maternal embryonic leucine zipper kinase (MELK), that is a member of the AMPK serine/threonine kinase family and is involved in the mammalian embryonic development[8], to be a promising drug target molecule for breast cancer[9]. MELK was also overexpressed in various types of human cancer including TNBC and its expression was hardly detectable in normal tissues except the testis[9-11]. In addition to the involvement in cancer cell growth, MELK was also reported its critical roles in formation or maintenance of cancer stem cells[12, 13], that have the ability to self-renew and differentiate. Emerging evidence indicated that the cancer stem cells are resistant to chemotherapy and radiation therapy, and are associated with the cancer relapse[14, 15]. Thus, targeting cancer stem cell is considered as a novel strategy for cancer treatment[16, 17]. The mechanisms how cancer cells acquired these abilities are not yet understood, but recent studies indicated that MELK is one of the marker molecules to characterize cancer stem cells in tumor, such as breast cancer and glioblastoma[13, 18]. Thus, targeting MELK could be an effective strategy to treat multiple types of human cancer.

In this study, we report development of a small-molecule MELK inhibitor OTSSP167 that can selectively and effectively inhibit MELK kinase activity. Our *in vitro* and *in vivo* studies also imply that OTSSP167 significantly suppresses mammosphere formation of breast cancer cells as well as the growth of human cancer-derived xenografts in mice, implying that OTSSP167 has great potential to apply as a novel therapeutics for cancer in a MELK-dependent manner. Furthermore, to verify the molecular mechanism of this MELK-specific inhibitor, we demonstrate identification of new substrates of MELK and inhibitory effect of the compound on activities of these molecules in breast cancer cells.

## RESULTS

### High-through put screening to identify MELK-specific inhibitor

**Figure 1:**
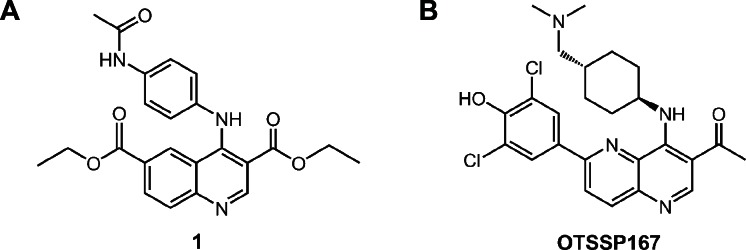
Novel MELK inhibitors (A) A quinoline derivative, diethyl 4-(4-acetamidophenylamino) quinoline-3,6-dicarboxylate (compound 1; a commercially available compound), was found to have a moderate inhibitory activity against MELK (IC_50_ = 4.8 μM) through the high-throughput screening. The subsequent structure-activity relationship study led to the synthesis of a highly potent MELK inhibitor with a novel structure: (B) compound OTSSP167, 1-(6-(3,5-dichloro-4-hydroxyphenyl)-4-((*trans*-4-((dimethylamino)methyl)cyclohexyl)amino)-1,5-naphthyridin-3-yl)ethanone. The dihydrochloride salt form was used in experiments for OTSSP167.

To obtain small-molecule MELK inhibitors, we first conducted high-throughput screening of a library consisting of 108,269 compounds. Each compound was screened at a single concentration of 30 μM against MELK using the IMAP assay[19] optimized for the high-throughput low-volume 384-well format assays (see Supplementary Methods). The inhibition activity was measured by percent of inhibition of the MELK kinase activity relative to control. The average and standard deviation of the percent inhibition were 0.87% and 9.07%, respectively. A total of 597 compounds revealed the MELK kinase inhibitory activity by 37.1% or higher. After validation by dose-response analysis, a quinoline derivative (compound 1 in Fig 1A) was confirmed to inhibit the MELK activity with the half-maximum inhibitory concentration (IC_50_) value of 4.8 μM. To develop high-affinity MELK inhibitors, we performed an intensive structure-activity relationship study on the basis of the structure of compound 1, and obtained novel compounds with various degrees of MELK inhibitory activity. Among them, the compound OTSSP167 (Fig 1B) was identified as one of the most effective MELK inhibitor with IC_50_ value of 0.41 nM (see Supplementary Methods for the compound synthesis and the kinase assay). OTSSP167 has a 1,5-naphthyridine core with methylketone at the 3-position, *trans*-4-((dimethylamino)methyl) cyclohexylamino at the 4-position, and 3,5-dichloro-4- hydroxyphenyl at the 6-position of the core.

### Growth suppressive effect of OTSSP167 in various cancer types

**Figure 2:**
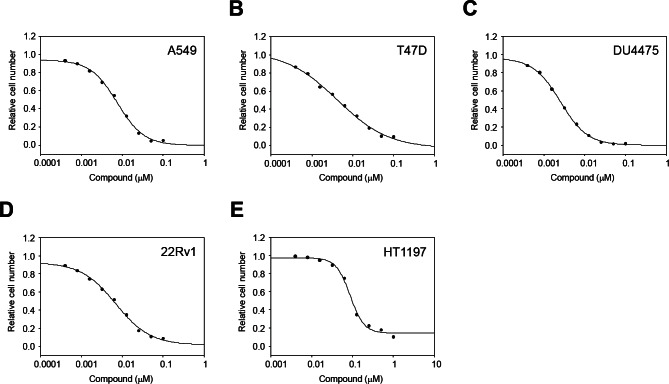
In vitro anti-proliferative activity of OTSSP167 The graphs indicate growth inhibition curves of OTSSP167 for various types of human cancel cell line; (A) A549 (lung cancer), (B) T47D (breast cancer), (C) DU4475 (breast cancer), and (D) 22Rv1 (prostate cancer) cells, in which MELK is highly expressed, as well as (E) HT1197 (bladder cancer) cell line, in which MELK expression is hardly detectable.

Since MELK was reported to be overexpressed in other types of human cancer in addition to breast cancer[9, 10], we examined the growth inhibitory effect of OTSSP167 on the growth of various cancer cell lines. *In vitro* anti-proliferative assay using A549 (lung), T47D (breast), DU4475 (breast), and 22Rv1 (prostate) cancer cells, in which MELK was highly expressed, revealed IC_50_ values of 6.7, 4.3, 2.3, and 6.0 nM, respectively (Fig 2A-D). On the other hand, HT1197 (bladder) cancer cells, in which MELK expression was hardly detectable, revealed IC_50_ value of 97 nM (Fig 2E), clearly implying the MELK-dependent growth-inhibition effect of this compound.

### Growth suppressive effect of OTSSP167 in xenograft mouse model

**Figure 3:**
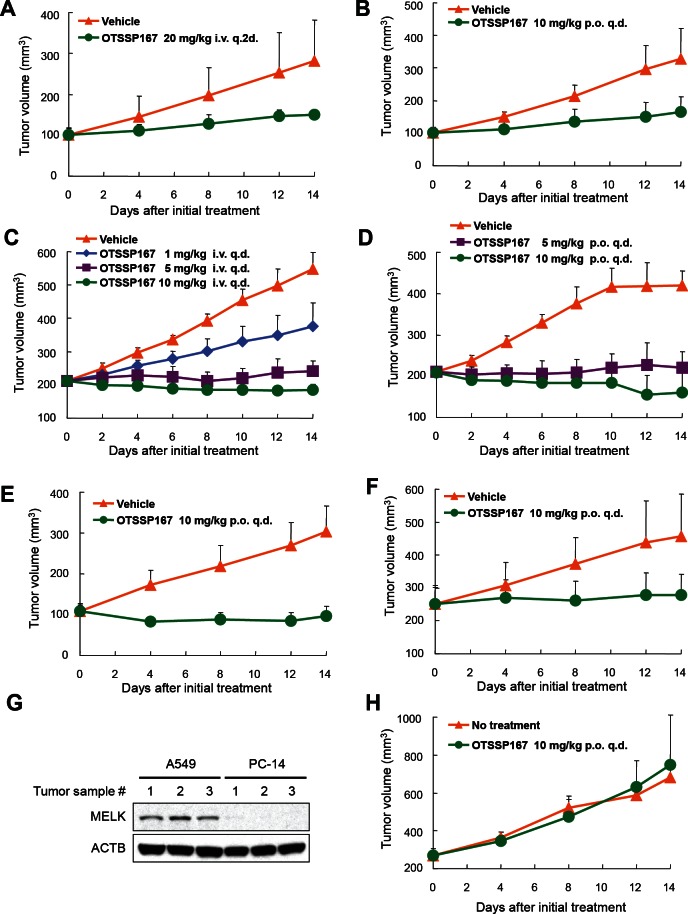
Mice xenograft models showing the effectiveness of OTSSP167 on the growth of various human cancer xenograft Nude mice bearing (A,B) MDA-MB-231 (triple-negative breast cancer), (C,D) A549 (lung cancer), (E) DU145 (prostate cancer), or (F) MIAPaCa-2 (pancreatic cancer) were treated with either vehicle control or OTSSP167 of given concentrations for 14 days. The administration doses were (A) 20 mg/kg intravenously once every two days or (B) 10 mg/kg orally once a day for MDA-MB-231; (C) 1, 5, or 10 mg/kg intravenously once a day or (D) 5 or 10 mg/kg orally once a day for A549; (E) 10 mg/kg orally once a day for DU145; and (F) 10 mg/kg orally once a day for MIAPaCa-2. Mean tumor volumes ± SD (n = 6 for each treatment group) are shown. (G) Lysates of tumor samples taken from A549 and PC-14 xenograft mice were immunoblotted with anti-MELK and anti-ACTB antibodies. (H) OTSSP167 was administered to nude mice bearing PC-14 (MELK-negative bladder cancer cells) at a dose of 10 mg/kg orally once a day. Mean tumor volumes ± SD (n = 3 per group) are shown. i.v. q.2d; intravenously once every two days, i.v. q.d.; intravenously once a day, p.o. q.d.; orally once a day.

We subsequently investigated *in vivo* anti-tumor effect of OTSSP167 by a xenograft model using MDA-MB-231 cells (MELK-positive, triple-negative breast cancer cells). The compound was administered to mice bearing xenografts for 14 days after the tumor size reached about 100 mm^3^. The tumor size was measured as a surrogate marker of drug response (tumor growth inhibition (TGI)). Intravenous administration of OTSSP167 at 20 mg/kg once every two days resulted in TGI of 73% (Fig 3A). Since the bioavailability of this compound was expected to be very high (data not shown), we attempted oral administration of this compound. The oral administration at 10 mg/kg once a day revealed TGI of 72% (Fig 3B). Due to the strong growth-suppressive effect on various cancer cell lines, we further investigated *in vivo* growth-suppressive effect using cancer cell lines of other types and found significant tumor growth suppression by OTSSP167 for multiple cancer types in dose-dependent manners with no or a little body-weight loss (Fig 3 and Supplementary Fig. S1). For example, mice carrying A549 (lung cancer) xenografts that were treated with 1, 5, and 10 mg/kg once a day of OTSSP167 by intravenous administration revealed TGI of 51, 91, and 108%, respectively (Fig 3C) and those by oral administration of 5 and 10 mg/kg once a day revealed TGI of 95 and 124%, respectively (Fig 3D). In addition, we examined DU145 (prostate cancer) and MIAPaCa-2 (pancreatic cancer) xenograft models by oral administration of 10 mg/kg once a day, and observed TGI of 106 and 87%, respectively (Fig 3E and F). To further validate the MELK-specific *in vivo* tumor suppressive effect, we examined PC-14 lung cancer cells in which MELK expression was hardly detectable (Fig 3G). Oral administration of 10 mg/kg OTSSP167 once a day for 14 days showed no tumor growth suppressive effect on PC-14 xenografts (Fig 3H), further supporting the MELK-dependent antitumor activity of OTSSP167.

### OTSSP167 inhibits the phosphorylation of novel MELK substrates

**Figure 4:**
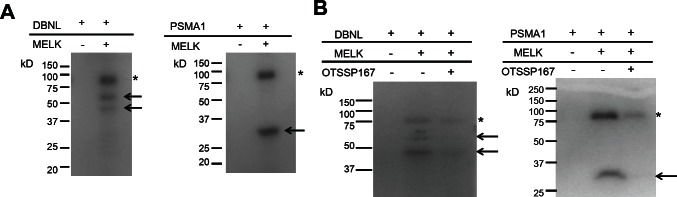
Identification and functional analysis of MELK substrates in breast cancer cell lines (A) *In vitro* kinase assay using recombinant proteins confirmed DBNL and PSMA1 to be novel substrates of MELK. Arrows indicate phosphorylated substrate proteins; asterisks indicate autophosphorylated MELK. (B) *In vitro* kinase assay using OTSSP167. DBNL (55kDa) or PSMA1 (30kDa) recombinant protein was incubated with MELK with or without OTSSP167. Asterisks indicate autophosphorylated MELK; arrows indicate phosphorylated substrates. Phopshorylation of each substrate was diminished by addition of 10 nM of OTSSP167

To further characterize the molecular mechanism of MELK overexpression in mammary carcinogenesis and validate the functional consequence of small molecule inhibitor against MELK, we further investigated MELK substrates. Using the MELK recombinant protein, we performed *in vitro* kinase assay in combination with 2D-PAGE and identified multiple candidate spots which appeared in a MELK-specific manner. We analyzed these spots by mass spectrometry and confirmed drebrin-like (DBNL) and proteasome subunit alpha type 1 (PSMA1) to be MELK substrates by *in vitro* kinase assay using their recombinant proteins as shown in Fig 4A. We subsequently performed *in vitro* kinase assay with these two substrates to confirm the phosphorylation-inhibitory effect of OTSSP167. As shown in Fig 4B, addition of this compound in an *in vitro* kinase assay significantly suppressed the phosphorylation levels of DBNL and PSMA1, further supporting strong inhibitory effect of this compound on the MELK activity.

### Phosphorylated DBNL by MELK enhances cellular invasiveness in cancer cell

**Figure 5:**
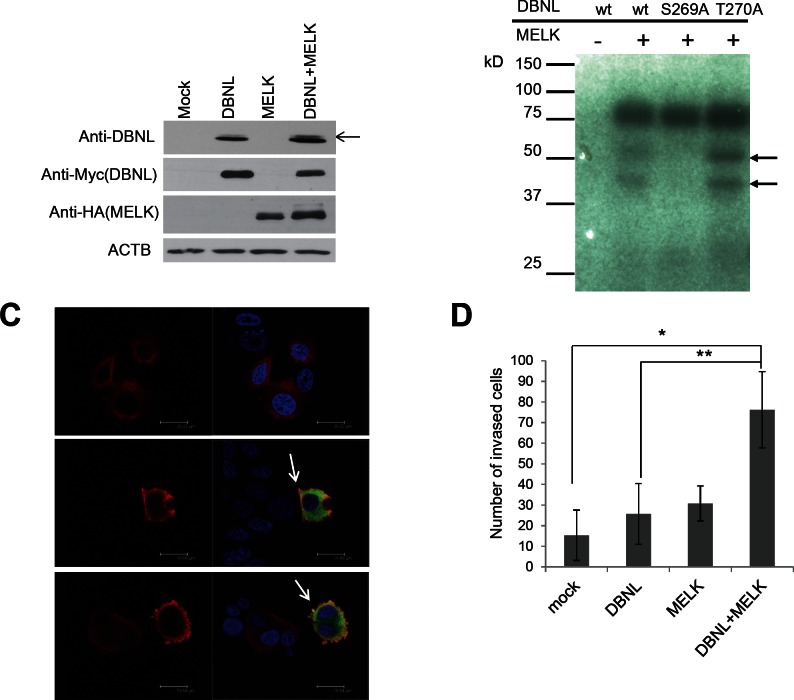
MELK phosphorylated Ser269 on DBNL and induced the cellular invasiveness (A) *In vivo* phosphorylation assay. Phosphorylation of DBNL (indicated by an arrow) in BT549 cells in which DBNL and MELK were co-transfected was enhanced much stronger than that in the cells in which only DBNL was transfected. (B) Identification of phosphorylated sites by *in vitro* kinase assay. Amino acid substituted mutants of DBNL were generated and used for *in vitro* kinase assay. Phosphorylated band of DBNL in which a serine 269 was substituted with an alanine (S269A) was completely diminished, while that of DBNL in which a threonine 270 was substituted with an alanine (T270A) was unchanged. wt; wild-type. Closed arrows indicate phosphorylated DBNL. (C) Immunocytochemical analysis of cells overexpressing DBNL with/without MELK. MCF-7 cells in which both MELK and DBNL were over-expressed shows a strongly enhanced membrane-ruffling pattern (Red; DBNL, Green; MELK, Blue; DAPI) which is indicated by white arrow. (D) MCF-7 cells over-expressing DBNL revealed elevated cell invasiveness in the presence of MELK. The number of invaded cells on Y-axis indicates the average cell number of migration, that was counted by microscopic observation (*p=0.009, **p=0.0209, student's t-test). Error bars represent means ± SD of triplicates.

DBNL is known to be an actin-binding adaptor protein that regulates the actin cytoskeleton and endocytosis[20-22]. To characterize the biological function of DBNL in human cancer, we first examined *in vivo* phosphorylation status of DBNL by western blot analysis using BT549 cells treated with Okadaic acid that can inhibit the phosphatase activity[23]. We introduced either or both of MELK and DBNL expression vectors into the cells, and detected the significant elevation of DBNL phosphorylation in the cells trasnfected with both expression vectors, compared with the cells transfected with either of the genes or the mock vector (Fig 5A). In addition, to identify the phosphorylation sites of DBNL by MELK, we performed mass spectrometry analysis in the presence or absence of MELK, and identified and confirmed Ser269 as a candidate phophorlylation site as shown in Fig 5B; the substitution of Ser269 of DBNL with an alanine completely diminished the phosphorylation by MELK, while that of Thr270 with an alanine showed no effect on the phosphorylation status. Subsequently, immunocytochemical analysis revealed drastic enhancement of membrane ruffling of the cells that were co-transfected with both DBNL and MELK (Fig 5C). Since membrane ruffling is related to tumor cell mobility and cancer metastasis[24], we performed Matrigel invasion assay (Fig 5D) and observed significantly higher invasiveness of the cells overexpressing both DBNL and MELK than the control cells or those overexpressing either of the two genes.

### OTSSP167 suppresses mammosphere formation through the inhibition of PSMA1 phosphorylation

**Figure 6:**
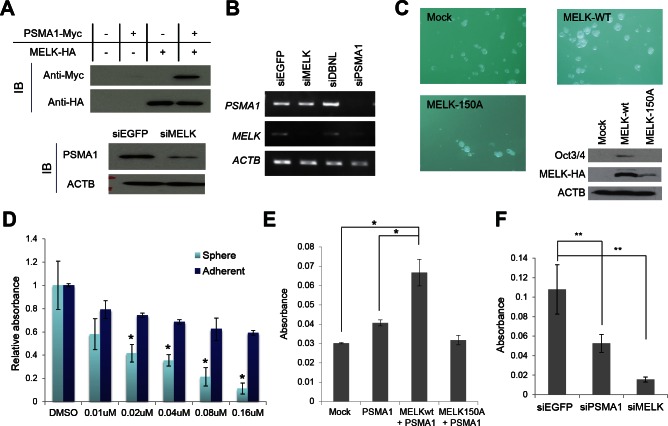
PSMA1 enhanced the mammosphere formation through the phosphorylation by MELK (A, B) PSMA1 protein was stabilized through the phosphorylation by MELK in breast cancer cells (A) although transcriptional level of PSMA1 was unchanged in the cells in which MELK expression was knocked down (B). (C) Wild-type MELK (MELK-wt) or kinase-dead mutant MELK (MELK-150A) expression vector was transfected into MCF-7 cells which were seeded onto an ultra-low attachment culture plate. The formation of mammosphere was enhanced in cells in which MELK-wt was transiently introduced than those transfected with mock vector or MELK-150A. The expression levels of one of cancer stem cell markers, Oct3/4, are shown. The cells which transiently over-expressed MELK-wt induced Oct3/4 expression while those transfected with mock vector or MELK-150A revealed no Oct3/4 expression. (D) OTSSP167 suppressed more significantly the formation of mammosphere than the growth of attached MCF-7 cells. The cells were plated onto ultra-low attachment culture plate or normal culture plate without or with OTSSP167 of given concentrations (*p<0.05, student's t-test). (E) The MCF-7 cells in which both PSMA1 and wild-type MELK (MELK wt) were co-overexpressed revealed higher number of mammosphere formation than the parental MCF-7 cells or those transfected with PSMA1 alone or PSMA1 + kinase-dead MELK (MELK D150A) (*p<0.0001, student's t-test). (F) The mammosphere formation of MDA-MB-231 cells, in which PSMA1 was knocked down, was suppressed (**p<0.05, student's t-test). Absorbance measured at 490 nm is indicated using that at 630 nm as a reference with a microplate reader. Error bars represent means ± SD of triplicates for experiments D-F.

PSMA1 is a subunit of the proteasome complex and was reported to be upregulated in breast cancer cells[25]. To examine the biological effect of MELK on PSMA1, we trasnfected either or both of MELK and PSMA1 into BT549 cells, and found the increase of the PSMA1 protein when PSMA1 was co-transfected with MELK, compared with the cells transfected with PSMA1 alone (Fig 6A). In concordance with this result, when we knocked-down MELK in T47D cells using siRNA, the amount of PSMA1 protein was drastically reduced, compared with the parental cells or the cells treated with control siRNA (Fig 6A), while the amount of PSMA1 transcript was unchanged (Fig 6B). These results have indicated that MELK possibly stabilizes PSMA1 protein through its phosphorylation. Since the knockdown of PSMA1 expression suppressed the proliferation of cancer cells (data not shown), the PSMA1 is also considered to be essential for survival of cancer cells. Previous studies suggested contribution of MELK in cancer stem cells due to its high level of expression in cancer stem cell populations (ex, CD133-positive glioblastoma cells)[10, 12, 13]. Our results in Fig 6C also revealed that upregulation of MELK promoted the mammosphere formation of breast cancer cells and induced the Otc3/4 expression that is well known as one of the stem cell markers while that of kinase-dead MELK (D150A) did not. Moreover, in mammosphere formation assay using MCF-7 breast cancer cells, the cells that were treated with OTSSP167 revealed stronger inhibition in its mammosphere formation than in the growth of adherent cells (Fig 6D), suggesting that OTSSP167 is likely to suppress effectively the growth of cancer stem cells. Interestingly, overexpression of PSMA1 was reported to play critical roles in hematopoietic stem progenitor cells[26]. Hence, we investigated possible involvement of PSMA1 phosphorylation by MELK in the maintenance of cancer stem cell characteristics. We performed mammosphere formation assay using MCF-7 cells which transiently over-expressed PSMA1 with either wild-type MELK or kinase-dead MELK, and found co-overexpression of PSMA1 and wild-type MELK strongly enhanced sphere formation, compared with the parental MCF-7 cells or those transfected with PSMA1 or PSMA1+ kinase-dead MELK (Fig 6E). Concordantly, the depletion of PSMA1 or MELK expression in MDA-MB-231 cells using siRNA significantly suppressed the formation of mammosphere (Fig 6F). Taken together, these results suggest that OTSSP167 suppressed mammosphere formation of cancer stem cells through the reduction of phosphorylated PSMA1 by inhibition of the MELK activity.

## DISCUSSION

After a great success of Imatinib in the treatment of chronic myelogenous leukemia (CML) and gastrointestinal stromal tumors (GISTs), many scientists and industries have been focusing on the development of drugs targeting on cancer-specific molecules[27]. Protein kinases are considered as attractive therapeutic targets for development of anti-cancer drugs because they play critical roles in growth-signaling pathways in cancer cells[28-31]. However, development of an inhibitor(s) which specifically suppresses target kinase activity is not so easy because most of kinase inhibitors are type 1 inhibitor which recognizes an ATP-pocket highly conserved across kinases and competes with ATP. These structural conservation leads to the unexpected cross-reactivity, in some cases yielding unexpected and unfavorable side effects[28, 32]. For discovering new kinase inhibitors with high effectiveness and minimum toxicity, the combination of identification of appropriate target molecules coupled with advanced drug-development tools including analogue synthesis, structure-informed design and fragment-based assembly is essential[28, 33].

To develop MELK-specific inhibitors in this study, we conducted the high-throughput screening for identification of hit compounds and subsequent intensive structure-activity informed study, and finally developed OTSSP167 which effectively inhibited the MELK kinase activity with IC_50_ of 0.41 nM. We then investigated the effect of OTSSP167 on the formation of mammosphere, one of the characteristics of breast cancer stem cells since MELK was reported as a key molecule for cancer stem-cell formation/maintenance[13]. Our results showed that OTSSP167 inhibited mammosphere formation in a dose-dependent manner and also revealed strong growth-suppressive effect on various types of human cancer xenograft including breast, pancreas, prostate and lung cancers without no or a little body-weight loss at the effective doses. The experiment using the MELK-negative cancer cells supported the MELK-dependent growth suppressive effect of OTSSP167 on cancer cells.

In parallel, to further characterize biological mechanisms of the MELK-signaling pathway and verify the mode of action of the MELK inhibitor OTSSP167, we screened novel MELK substrates and identified two possible candidate molecules, DBNL and PSMA1. DBNL is a member of the debrin/Abp1 family of actin-binding proteins and is a component of the immunological synapse that regulates T-cell activation[34]. Although there was no evidence of DNBL involvement in human carcinogenesis, our data have indicated that the phosphorylation of DBNL by MELK is likely to promote cancer cell invasiveness, and probably lead to tumor recurrence and poor prognosis[35]. We also found that MELK could phosphorylate Ser269 on DBNL. Since the phosphrylation of this site was reported to be critical to bind to 14-3-3 proteins[36] that has important roles in the regulation of numerous cellular signaling pathways like cell cycle regulation or apoptosis[37], we suspect that MELK might promote cell growth and mobility of cancer cells through the regulation of the DBNL-14-3-3 signaling pathway.

The other substrate, PSMA1, is one of the components of the 20S core structure of proteasome complex that is important to regulate the concentration of intracellular proteins and remove misfolded proteins through degrading them[38]. The function of PSMA1 itself was not well understood, however, its phosphorylation might affect the assembly of the proteasome complex[39]. A recent study suggested that enhancement of the proteasome assembly and activity could play crucial roles in the maintenance of human embryonic stem cells[40]. We also investigated the biological characteristics of PSMA1 in cancer cells, and found that PSMA1 was stabilized by the phosphorylation in MELK overexpressing cells and that coexistence of PSMA1 and MELK enhanced the formation of mammosphere. Interestingly, the number of mammosphere was significantly decreased in the cells in which PSMA1 expression was knocked down by siRNA for PSMA1. Our data imply that OTSSP167 possibly suppressed mammosphere formation through the reduction of PSMA1 protein.

In summary, we have demonstrated that MELK plays crucial roles in cancer progression and/or stem cell maintenance through phosphorylation of its substrate proteins. Our data have also indicated that that the selective MELK inhibitor OTSSP167 could suppress the phosphorylation of these two MELK substrates, and has the *in vitro* and *in vivo* growth suppressive effect on cancer cells, implicating a great potential of this MELK inhibitor to apply to treatment of various types of human cancer.

## METHODS

### High-throughput screening

A library consisting of 108,269 compounds (AMRI's Diverse AMRI Synthetic Library (DASL)) was screened using the assay protocol optimized for the high-throughput low-volume 384-well format assays. Each of the compounds (30 μM) in 342 library plates were incubated for 120 min at room temperature, with 70 μM of ATP, 100 nM of the substrate peptide, and 30 nM of MELK protein. Any plate that showed Z’<0.5 was retested (more details in Supplementary Methods).

### Cell lines, plasmids, oligo siRNAs and transfection

MCF-7, MDA-MB-231, BT549, T47D, DU4475, 22Rv1, DU145, HT1197, and NIH3T3 cells were purchased from the American Type Culture Collection (ATCC) (Rockville, MD, USA). A549, PC-14, and MIAPaCa-2 cells were purchased from European Collection of Cell Cultures (ECACC) (Salisbury, UK), RIKEN BioResource Center (Tsukuba, Japan), and Japanese Collection of Research Bioresources Cell Bank (JCRB) (Suita, Japan), respectively. All cells were cultured under appropriate media recommended by suppliers with 10% FBS and 1% antibiotic-antimycotic solution (Sigma-Aldrich). All cells except MDA-MB-231 were maintained at 37 °C in humidified air with 5% CO_2_. MDA-MB-231 was maintained at 37 °C in humidified air without CO_2_. MELK wild-type and kinase-dead mutant (D150A) plasmids were constructed previously[9]. To construct vectors designed to express DBNL (NM_001014436.2) or PSMA1 (NM_002786.3), the entire coding sequences were amplified by RT-PCR and cloned into the pcDNA3.1-myc-his or pCAGGSnHc expression vector. We carried out site-directed mutagenesis PCR to generate DBNL substituted mutants (S269A and T270A) with a QuickChange site-directed Mutagenesis kit (Stratagene). Plasmids were transfected using Fugene6 (for NIH3T3) or FugeneHD (for human breast cancer cell lines) (Roche) according to the supplier's recommendations. For knockdown experiments, cells were transfected with oligo siRNA using Lipofectamine RNAiMAX (Invitrogen) according to manufacturer's instructions. The target sequences of oligo siRNAs were as follows: 5'-GACAUCCUAUCUAGCUGCA-3' for MELK; 5'-CAGAUACCAACACAACGAU-3' for PSMA1; 5'-GGTGCTGGCTCTGAGCACA-3' for DBNL; 5′-TTGAAGCAGCACGACUUCUUC-3′ and 5′-TTGAAGAAGUCGUGCUGCUUC-3′ for siEGFP.

### Recombinant proteins and in vitro kinase assay for substrate screening

MELK recombinant protein was generated as described previously[9]. The full coding sequence of each of MELK substrate candidates was amplified by RT-PCR and cloned into the pGEX6p-1 vector (GE Healthcare). The GST-tagged recombinant proteins were expressed in BL21 codon-plus RIL competent cells (Stratagene) and purified using Glutathione Sepharose 4B beads (GE Healthcare) according to the supplier's instructions. The GST-tag was removed by PreScission protease (GE Healthcare) according to the supplier's instructions. For *in vitro* kinase assay, MELK recombinant protein (0.4 μg) was mixed with 5 μg of each substrate in 20 μl of kinase buffer containing 30 mM Tris-HCl (pH), 10 mM DTT, 40 mM NaF, 10 mM MgCl_2_, 0.1 mM EGTA with 50 μM cold-ATP and 10 Ci of [γ-^32^P]ATP (GE Healthcare) for 30 min at 30 °C. The reaction was terminated by addition of SDS sample buffer and boiled for 5 min prior to SDS-PAGE. The gel was dried and autoradiographed with intensifying screens at room temperature. OTSSP167 (final concentration of 10 nM) was dissolved in DMSO and added to kinase buffer before the incubation.

### Western blot analysis and immunocytochemistry

Cells were lysed with RIPA buffer containing protease inhibitor cocktail and phosphatase inhibitor cocktail (Calbiochem). The proteins were separated by electrophoresis using 10% or 7.5% SDS-PAGE gel and transferred onto nitrocellulose membrane. The membranes were incubated with the first antibody, respectively: anti-PSMA1 antibody (Epitomics), anti-DBNL antibody, anti-Myc (Santa Cruz Biothechnology), anti-HA (Roche), anti-Oct3/4 (Santa Cruz Biothechnology) or anti-ACTB. We generated mouse anti-MELK monoclonal antibodies using partial recombinant MELK protein (264-601 amino acids of MELK) as an immunogen by the methods as described previously[41]. For immunocytochemistry, MCF-7 cells were seeded onto glass slide-chamber and transfected with expression vector(s) as described above. After 48 hours of incubation, cells were fixed with 4% paraformaldehyde and permeabilized with 0.1% Triton X-100 in PBS for 1 min at room temperature. Non-specific binding was blocked by treatment with PBS containing 3% bovine serum albumin (BSA) for 30 min at room temperature. Cells were incubated for 60 min at room temperature with anti-HA or anti-DBNL antibody diluted at 1:200 by PBS containing 3% BSA. After washing with PBS, cells were stained by Alexa fluor-conjugated secondary antibody (Invitrogen) for 60 min at room temperature, and visualized with Spectral Confocal Scanning Systems (Leica).

### In vivo phosphorylation assay

DBNL expression vector was transfected into cells with or without MELK expression vector. After 48 hours of incubation, cells were treated with 100 nM Okadaic acid (Calbiochem) and incubated for 6 hours. The cells were lysed after the treatment with Okadaic acid and the lysed samples were loaded into 7.5% SDS-PAGE gel. The proteins were transferred onto nitrocellulose membrane (GE Healthcare). The membrane was incubated with anti-DBNL antibody (Abnova) or anti-β-actin (ACTB) (Sigma-Aldrich). ACTB served as a loading control.

### Matrigel invasion assay and mammosphere formation assay

NIH3T3 cells transfected with plasmids expressing MELK (pCAGGSnHc-MELK), DBNL (pcDNA3.1-Myc-His-DBNL) or both were grown to near confluence in DMEM containing 10% FBS. After the incubation of 24 hours, the cells were harvested by trypsinization, washed in DMEM without addition of serum, and suspended in serum-free DMEM. The cells (1'10^4^ cells) were seeded onto the Matrigel matrix chamber (BD Biosciences) and incubated for 22 hours. The cells invading to Matrigel were stained by Giemsa (Merck) and counted. For sphere formation assay, 1'10^3^ cells of MCF-7 cells which transiently over-expressed wild-type MELK, kinase-dead MELK, PMSA1, PSMA1 and wild-type MELK, or PMSA and kinase-dead MELK were seeded onto Ultra-Low attachment plate (Corning). For knockdown experiments, MDA-MB-231 cells (1'10^3^ cells) which seeded onto Ultra-Low attachment plate were transfected with oligo siRNA for EGFP, MELK or PSMA1 as described above. For examination of sphere formation under treatment of MELK inhibitor OTSSP167, 1'10^3^ MCF-7 cells were seeded with 0.01, 0.02, 0.04, 0.08, or 0.16 μM of OTSSP167, respectively. DMSO alone was used as a control. Following incubation for two weeks, cell viability was measured by using Cell-counting kit-8 (DOJINDO).

### In vivo xenograft study

MDA-MB-231 cells were injected into the mammary fat pads of NOD.CB17-*Prkdc^scid^*/J mice (Charles River Laboratory). A549, MIAPaCa-2 and PC-14 cells (1 × 10^7^ cells) were injected subcutaneously in the left flank of female BALB/cSLC-nu/nu mice (Japan SLC, Inc.). DU145 cells were injected subcutaneously in the left flank of male BALB/cSLC-nu/nu mice (Japan SLC, Inc.). When MDA-MB-231, A549, DU145, MIAPaCa-2, and PC-14 xenografts had reached an average volume of 100, 210, 110, 250, and 250 mm^3^, respectively, animals were randomized into groups of 6 mice (except for PC-14, for which groups of 3 mice were used). For oral administration, compounds were prepared in a vehicle of 0.5% methylcellulose and given by oral garbage at the indicated dose and schedule. For intravenous administration, compounds were formulated in 5% glucose and injected into the tail vein. An administration volume of 10 ml per kg of body weight was used for both administration routes. Concentrations were indicated in main text and Figures. Tumor volumes were determined every other day using a caliper. The results were converted to tumor volume (mm^3^) by the formula length × width^2^ × 1/2. The weight of the mice was determined as an indicator of tolerability on the same days. The animal experiments using A549 xenografts were conducted by contract with KAC Co., Ltd. (Shiga, Japan) in accordance with their Institutional Guidelines for the Care and Use of Laboratory Animals. The other animal experiments were conducted at OncoTherapy Science, Inc. in accordance with their Institutional Guidelines for the Care and Use of Laboratory Animals. Tumor growth inhibition (TGI) was calculated according to the formula {1 – (*T* – *T*_0_) / (*C* – *C*_0_)}×100, where *T* and *T*_0_ are the mean tumor volumes at day 14 and day 0, respectively, for the experimental group, and *C* – *C*_0_ are those for the vehicle control group.

### Statistical analysis

All values were presented as means ± SD. Statistical significance was computed using student's t-test, and the level of significance was set at p<0.05.

Detailed methods are described in the Supplementary Methods.

## Supplementary Materials



## References

[1] Jemal A, Siegel R, Ward E, Hao Y, Xu J, Murray T, Thun MJ (2008). Cancer statistics, 2008. CA Cancer J Clin.

[2] Forouzanfar MH, Foreman KJ, Delossantos AM, Lozano R, Lopez AD, Murray CJ, Naghavi M (2011). Breast and cervical cancer in 187 countries between 1980 and 2010: A systematic analysis. Lancet.

[3] Sotiriou C, Pusztai L (2009). Gene-expression signatures in breast cancer. N Engl J Med.

[4] Rakha EA, Elsheikh SE, Aleskandarany MA, Habashi HO, Green AR, Powe DG, El-Sayed ME, Benhasouna A, Brunet JS, Akslen LA, Evans AJ, Blamey R, Reis-Filho JS, Foulkes WD, Ellis IO (2009). Triple-negative breast cancer: Distinguishing between basal and nonbasal subtypes. Clin Cancer Res.

[5] Griffiths CL, Olin JL (25). Triple negative breast cancer: A brief review of its characteristics and treatment options. J Pharm Pract.

[6] Cleator S, Heller W, Coombes RC (2007). Triple-negative breast cancer: Therapeutic options. Lancet Oncol.

[7] Foulkes WD, Smith IE, Reis-Filho JS (2010). Triple-negative breast cancer. N Engl J Med.

[8] Heyer BS, Warsowe J, Solter D, Knowles BB, Ackerman SL (1997). New member of the snf1/ampk kinase family, melk, is expressed in the mouse egg and preimplantation embryo. Mol Reprod Dev.

[9] Lin ML, Park JH, Nishidate T, Nakamura Y, Katagiri T (2007). Involvement of maternal embryonic leucine zipper kinase (melk) in mammary carcinogenesis through interaction with bcl-g, a pro-apoptotic member of the bcl-2 family. Breast Cancer Res.

[10] Gray D, Jubb AM, Hogue D, Dowd P, Kljavin N, Yi S, Bai W, Frantz G, Zhang Z, Koeppen H, de Sauvage FJ, Davis DP (2005). Maternal embryonic leucine zipper kinase/murine protein serine-threonine kinase 38 is a promising therapeutic target for multiple cancers. Cancer Res.

[11] Bianchini G, Iwamoto T, Qi Y, Coutant C, Shiang CY, Wang B, Santarpia L, Valero V, Hortobagyi GN, Symmans WF, Gianni L, Pusztai L (2010). Prognostic and therapeutic implications of distinct kinase expression patterns in different subtypes of breast cancer. Cancer Res.

[12] Sutter R, Yadirgi G, Marino S (2007). Neural stem cells, tumour stem cells and brain tumours: Dangerous relationships?. Biochim Biophys Acta.

[13] Hebbard LW, Maurer J, Miller A, Lesperance J, Hassell J, Oshima RG, Terskikh AV (2010). Maternal embryonic leucine zipper kinase is upregulated and required in mammary tumor-initiating cells in vivo. Cancer Res.

[14] Rich JN (2007). Cancer stem cells in radiation resistance. Cancer Res.

[15] Dean M, Fojo T, Bates S (2005). Tumour stem cells and drug resistance. Nat Rev Cancer.

[16] Kemper K, Grandela C, Medema JP (2010). Molecular identification and targeting of colorectal cancer stem cells. Oncotarget.

[17] Curtin JC, Lorenzi MV (2010). Drug discovery approaches to target wnt signaling in cancer stem cells. Oncotarget.

[18] Nakano I, Masterman-Smith M, Saigusa K, Paucar AA, Horvath S, Shoemaker L, Watanabe M, Negro A, Bajpai R, Howes A, Lelievre V, Waschek JA, Lazareff JA, Freije WA, Liau LM, Gilbertson RJ (2008). Maternal embryonic leucine zipper kinase is a key regulator of the proliferation of malignant brain tumors, including brain tumor stem cells. J Neurosci Res.

[19] Sportsman JR, Gaudet EA, Boge A (2004). Immobilized metal ion affinity-based fluorescence polarization (imap): Advances in kinase screening. Assay Drug Dev Technol.

[20] Larbolette O, Wollscheid B, Schweikert J, Nielsen PJ, Wienands J (1999). Sh3p7 is a cytoskeleton adapter protein and is coupled to signal transduction from lymphocyte antigen receptors. Mol Cell Biol.

[21] Mise-Omata S, Montagne B, Deckert M, Wienands J, Acuto O (2003). Mammalian actin binding protein 1 is essential for endocytosis but not lamellipodia formation: Functional analysis by rna interference. Biochem Biophys Res Commun.

[22] Kessels MM, Engqvist-Goldstein AE, Drubin DG, Qualmann B (2001). Mammalian abp1, a signal-responsive f-actin-binding protein, links the actin cytoskeleton to endocytosis via the gtpase dynamin. J Cell Biol.

[23] Cohen P, Holmes CF, Tsukitani Y (1990). Okadaic acid: A new probe for the study of cellular regulation. Trends Biochem Sci.

[24] Yamaguchi K, Hata K, Wada T, Moriya S, Miyagi T (2006). Epidermal growth factor-induced mobilization of a ganglioside-specific sialidase (neu3) to membrane ruffles. Biochem Biophys Res Commun.

[25] Deng S, Zhou H, Xiong R, Lu Y, Yan D, Xing T, Dong L, Tang E, Yang H (2007). Over-expression of genes and proteins of ubiquitin specific peptidases (usps) and proteasome subunits (pss) in breast cancer tissue observed by the methods of rfdd-pcr and proteomics. Breast Cancer Res Treat.

[26] He X, Gonzalez V, Tsang A, Thompson J, Tsang TC, Harris DT (2005). Differential gene expression profiling of cd34+ cd133+ umbilical cord blood hematopoietic stem progenitor cells. Stem Cells Dev.

[27] Zhang Z, Stiegler AL, Boggon TJ, Kobayashi S, Halmos B (2010). Egfr-mutated lung cancer: A paradigm of molecular oncology. Oncotarget.

[28] Zhang J, Yang PL, Gray NS (2009). Targeting cancer with small molecule kinase inhibitors. Nat Rev Cancer.

[29] Schmidt-Kittler O, Zhu J, Yang J, Liu G, Hendricks W, Lengauer C, Gabelli SB, Kinzler KW, Vogelstein B, Huso DL, Zhou S (2010). Pi3kalpha inhibitors that inhibit metastasis. Oncotarget.

[30] Liu-Sullivan N, Zhang J, Bakleh A, Marchica J, Li J, Siolas D, Laquerre S, Degenhardt YY, Wooster R, Chang K, Hannon GF, Powers S (2011). Pooled shrna screen for sensitizers to inhibition of the mitotic regulator polo-like kinase (plk1). Oncotarget.

[31] Hantschel O, Grebien F, Superti-Furga G (2011). Targeting allosteric regulatory modules in oncoproteins: «Drugging the undruggable». Oncotarget.

[32] Crespo A, Zhang X, Fernandez A (2008). Redesigning kinase inhibitors to enhance specificity. J Med Chem.

[33] Morachis JM, Huang R, Emerson BM (2011). Identification of kinase inhibitors that target transcription initiation by rna polymerase ii. Oncotarget.

[34] Le Bras S, Foucault I, Foussat A, Brignone C, Acuto O, Deckert M (2004). Recruitment of the actin-binding protein hip-55 to the immunological synapse regulates t cell receptor signaling and endocytosis. J Biol Chem.

[35] Friedl P, Locker J, Sahai E, Segall JE (2012). Classifying collective cancer cell invasion. Nat Cell Biol.

[36] Li X, Wolf ME (2011). Visualization of virus-infected brain regions using a gfp-illuminating flashlight enables accurate and rapid dissection for biochemical analysis. J Neurosci Methods.

[37] Hermeking H (2003). The 14-3-3 cancer connection. Nat Rev Cancer.

[38] Murata S, Yashiroda H, Tanaka K (2009). Molecular mechanisms of proteasome assembly. Nat Rev Mol Cell Biol.

[39] Bose S, Stratford FL, Broadfoot KI, Mason GG, Rivett AJ (2004). Phosphorylation of 20s proteasome alpha subunit c8 (alpha7) stabilizes the 26s proteasome and plays a role in the regulation of proteasome complexes by gamma-interferon. Biochem J.

[40] Vilchez D, Boyer L, Morantte I, Lutz M, Merkwirth C, Joyce D, Spencer B, Page L, Masliah E, Berggren WT, Gage FH, Dillin A (2012). Increased proteasome activity in human embryonic stem cells is regulated by psmd11. Nature.

[41] Fukukawa C, Hanaoka H, Nagayama S, Tsunoda T, Toguchida J, Endo K, Nakamura Y, Katagiri T (2008). Radioimmunotherapy of human synovial sarcoma using a monoclonal antibody against fzd10. Cancer Sci.

