# Supplementary material for: Development of an orally-administrative MELK-targeting inhibitor that suppresses the growth of various types of human cancer
Source: Oncotarget. 2012 Dec 21;3(12):1629–40. doi: 10.18632/oncotarget.790 (PMC3681500; doi:10.18632/oncotarget.790)
Supplement: Supplementary file 1 [file oncotarget-03-1629-s001.pdf]

Development of an orally-administrative MELK-targeting inhibitor that suppresses the growth of various types of human cancer – Chung et al

Supplementary Materials and Methods

2D-PAGE, silver staining and mass spectrometry. MELK recombinant protein (0.4 µg) was mixed with 5 µg of MCF7 whole cell lysate in 20 µl of kinase buffer containing 30 mM Tris-HCl (pH), 10 mM DTT, 40 mM NaF, 10 mM $MgCl_2$, 0.1 mM EGTA with 50 µM ATP for 30 min at 30 °C. After the incubation, samples were cleaned up by the 2D Clean-up kit (GE Healthcare) and the precipitate was dissolved in rehydration buffer (8 M urea, 4% CHAPS) with 0.5% immobilized pH gradient (IPG) buffer and 15l Destreak reagent (GE Healthcare). Subsequently the samples were centrifuged at 15,000rpm for 5 min to remove any insoluble material. One-hundred µg of protein was applied to 13 cm, pH3–10 NL Immobiline DryStrip (GE Healthcare). The strips were rehydrated overnight and the samples were resolved using Ettan IPGphor3 system (GE Healthcare). Following reduction and alkylation of the above strips, further separation of the proteins was carried out by SDS–PAGE using 10% SDS-PAGE gels. Following 2D-PAGE, the gels were stained with SilverQuest Silver Staining kit (Invitrogen). To identify phosphorylation sites, we performed *in vitro* kinase assay as described above and stained the gel using SimplyBlue SafeStain (Invitrogen). Bands were excised for mass spectrometry. The excised protein spots or bands were reduced in 10 mM tris(2-carboxyethyl)phosphine (Sigma) with 50 mM ammonium bicarbonate (Sigma) for 30 min at 37 °C and alkylated in 50 mM iodoacetamide (Sigma) with 50 mM ammonium bicarbonate for 45 min in the dark at 25 °C. Trypsin GOLD (Promega) solution was added with the enzyme to protein ratio at 1/50 (w/w) and incubated at 37 °C for 16 hours. The resulting peptides were extracted from gel fragments and separated on a 0.1 × 200 mm homemade C_{18} column using 30 min linear gradient from 5.4 to 29.2% acetonitrile in 0.1% formic acid with flow rate at 200 nl/min. The eluting peptides were analyzed with QSTAR Elite QqTOF system (AB Sciex) in the smart information-dependent acquisition (SIDA) mode of the Analyst QS software 2.0 (AB Sciex). The acquired MS peak lists were analyzed with in-house MASCOT server ver.2.3.01 (Matrix Science) to identify peptide sequences.

Recombinant MELK production and purification. To express a C-terminal His_6-tagged MELK, cDNA encoding human MELK was cloned into pET28a (+) vector (Novagen). The protein was expressed in *E. coli* BL21 (DE3) RIL strain (Stratagene) by addition of 0.2 mM isopropyl-1-thio-D-galactopyranoside and incubated overnight at 18 °C. The cells were harvested, resuspended in 20 mM phosphate (pH 7.4) supplemented with 500 mM NaCl, 40 mM imidazole, 1 mM dithiothreitol (DTT) and Complete Protease Inhibitor Cocktail (Roche), and lysed by sonication. The lysate was cleared by centrifugation at 9,400g for 10 min at 4 °C, and the supernatant was loaded onto a 5 mL HisTrap FF column (GE Healthcare). The column was washed with 10 column-volume of 20 mM phosphate (pH 7.4) supplemented with 500 mM NaCl, 40 mM imidazole and 1 mM DTT. The protein was eluted with 20 mM phosphate (pH 7.4) supplemented with 500 mM NaCl, 500 mM imidazole and 1 mM DTT, and loaded onto a Sephadex 75 HR 10/30 column (GE Healthcare) equilibrated with 50 mM phosphate (pH 7.4) containing 500 mM NaCl and 1 mM DTT. The protein was eluted in a set of fractions, desalted by HiPrep 26/10 desalting column (GE Healthcare) and loaded onto a 1 mL HiTrapQ HP column (GE Healthcare). The protein was eluted

with a 10 column-volume linear 50 mM to 1 M NaCl gradient, and dialyzed against 50 mM Tris-HCl (pH 7.5) containing 150 mM NaCl, 10 mM $MgCl_2$, 1 mM DTT and 10% glycerol.

Kinase assay for compound screening. Kinase activity was measured in the presence or absence of compounds using FAM-Glycogen Synthase-derived peptide (Molecular Devices Corporation) as a substrate. The extent of FAM-GS-derived peptide phosphorylation was measured by immobilized metal ion affinity-based fluorescence polarization (IMAP) technology[17] using IMAP FP Progressive Binding System (Molecular Devices Corporation). Test compounds were dissolved in DMSO at 12.5 mM and then serially diluted as the DMSO concentration in the assays to be 1%. The serially diluted compounds, 0.3 ng/µL MELK and 100 nM FAM-Glycogen Synthase-derived peptide were reacted in a reaction buffer (20 mM HEPES, 0.01% Tween-20, 0.3 mM $MgCl_2$, 2 mM dithiothreitol, 20 µM ATP, pH 7.4) at room temperature for 3 hours. The reaction was terminated by addition of three-fold assay volume of IMAP binding solution (Molecular Devices Corporation). Following 30 min incubation at room temperature, fluorescence polarization was measured by Wallac EnVision 2103 multilabel reader (PerkinElmer). IC_{50} values were calculated by nonlinear four parameter fit using SigmaPlot, version 10.0 (Systat Software, Inc.).

Cell-based assay. *In vitro* cell viability was measured by the colorimetric assay using Cell Counting Kit-8 (Dojindo Molecular Technologies, Inc.). Cells were plated in 100 µL in 96-well plates at a density that generated continual linear growth (A549, $1x10^3$ cells; T47D, $3x10^3$ cells; DU4475, $4x10^3$ cells; 22Rv1, $6x10^3$ cells; and HT1197, $2x10^3$ cells, in 100 µL per well). The cells were allowed to adhere overnight before exposure to compounds for 72 hours at 37 °C. Plates were read using a spectrophotometer at a wavelength of 450 nm. All assays were carried out in triplicate.

High-throughput screening. Liquid handling operations were performed using several instruments. Small volume transfers of 100 nL were performed using a Genomic Solutions Hummingbird 384-well non-contact nanoliter dispenser. Assays were initiated by sequential addition of reagents using an Aurora Discovery's BioRapTR FRD non-contact dispenser. Addition of IMAP binding reagent was performed using a Titertek Multidrop 384 liquid dispenser. A Beckman Coulter BioMek FX equipped with Span-8 was used for hit picking, and BioMek FX equipped with a 384-well head was used to prepare dose response source places. The fluorescent polarization was read using an Analyst GT multimode plate reader from Molecular Devices.The MELK kinase assay protocol was optimized for a low volume 384-well format for high-throughput screening. All assay plates had test compounds in columns 3-22, and controls in columns 1-2 and 23-24. The MAX (high signal or no inhibition) control wells received all assay components, including enzyme. The MIN (low signal) control wells received substrate and enzyme buffer but no enzyme. Based on preliminary concentration tests, the samples were tested at 30 µM against MELK. The standard operating procedure is outlined below: (1) Transfer library compounds resuspended in 100% DMSO into a dry 384-well low volume non-binding surface black polystyrene assay plate using the Hummingbird. Control wells receive equal volumes of 100% DMSO. (2) Dispense substrate followed by enzyme to the assay plate in a final volume of 5.0 µL using the BioRapTR. MIN control wells receive substrate followed by enzyme buffer without enzyme. (3) Shake plate on a plate shaker at high setting for 2 min. (4) Cover and incubate at room temperature for 120 min. (5) Dispense

10.0 µL IMAP binding reagent to all wells of the plate and incubate at room temperature for an additional 90 min. (6) Read plate on an Analyst GT with the following settings: Excitation, 485 nm; Emission, 530 nm; Dichroic, 505 nm; G Factor, 1; Z Height, 1 mm; Integration time, 0.1 s; and Attenuator, Out.

Synthesis of compound OTSSP167. The scheme of the synthesis of OTSSP167 is as shown below:

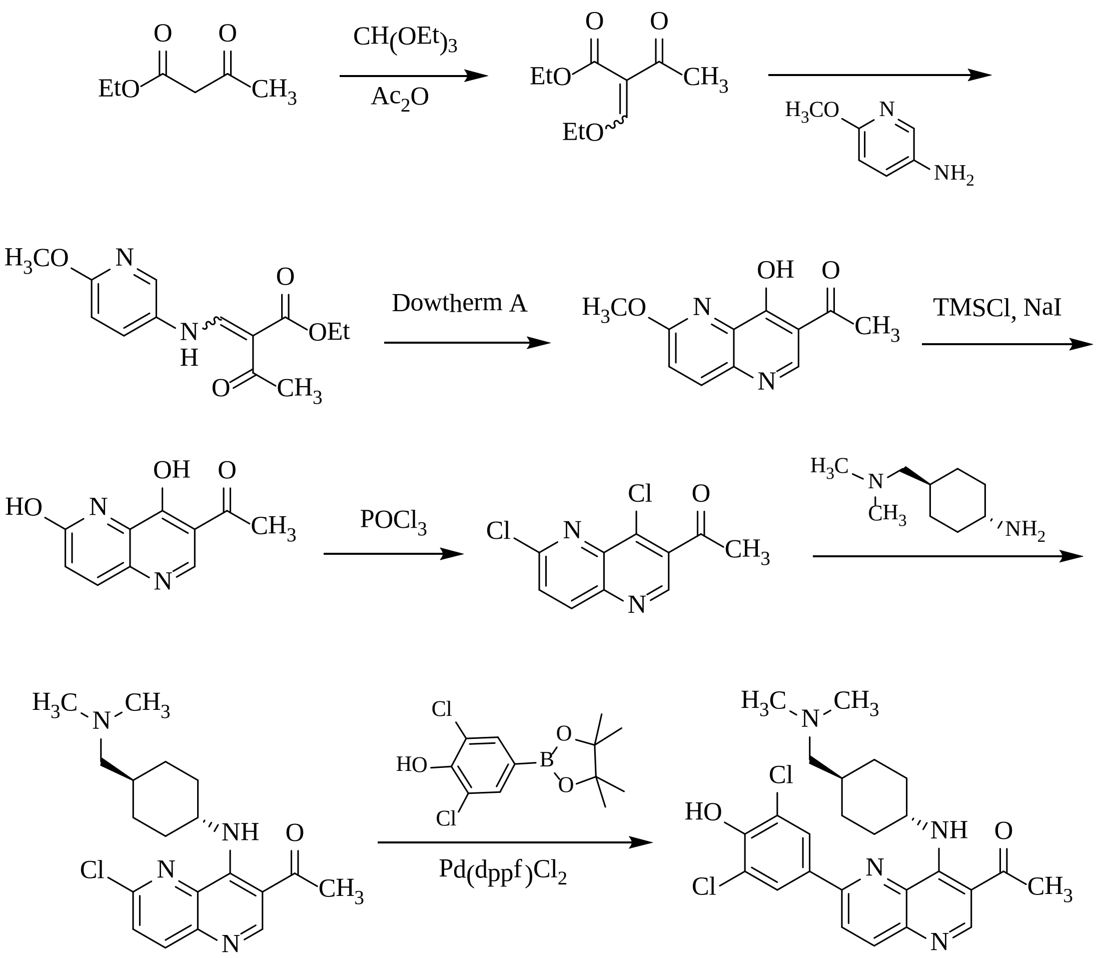

Ethyl 2-(ethoxymethylene)-3-oxobutanoate. To a 1 L round bottom flask equipped with a distillation apparatus was added ethyl 3-oxobutanoate (100 g, 0.77 mol), triethyl orthoformate (130 g, 0.92 mol), and acetic anhydride (150 g, 1.5 mol), and the reaction mixture was stirred with heating at 135 °C until the desired amount of ethanol was collected (~ 35 mL). The reaction was cooled, concentrated to remove all volatiles and the residue was distilled under high vacuum to obtain the desired product (100 g, 70%) as a pale yellow oil: ESI MS *m/z* 187 $[M + H]^+$.

Ethyl 2-((6-methoxypyridin-3-ylamino)methylene)-3-oxobutanoate. A mixture of ethyl 2-(ethoxymethylene)-3-oxobutanoate (100 g, 0.54 mol) and 2-methoxy-5- aminopyridine (67 g, 0.54 mol) in chlorobenzene (500 mL) was heated at 135 °C for 4 hours in a round bottom flask that was equipped with a distillation apparatus to collect the ethanol generated during the reaction. The reaction mixture was cooled and concentrated and the residue was triturated in diethylether and filtered to obtain the desired product (**3**, 120 g, 84%) as an off-white solid: ^{1}H NMR (500 MHz, $CDCl_3$) δ 12.74 (d, 12.3 Hz, 1H),

8.35 (d, J = 13.0 Hz, 1H), 8.07 (d, J = 2.8 Hz, 1H), 7.55 (d, J = 8.8, 2.9 Hz,, 1H), 6.79 (d, J = 8.8 Hz, 1H), 4.30 (q, J = 7.1 Hz, 2H), 2.55 (s, 3H), 1.33 (t, J = 7.1 Hz, 1H); ESI MS *m/z* 265 $[M + H]^+$.

1-(4-Hydroxy-6-methoxy-1,5-naphthyridin-3-yl)ethanone. A flask containing DowTherm A (500 mL) was heated to 240 – 250 °C and solid ethyl 2-((6-methoxypyridin-3-ylamino)methylene)-3-oxobutanoate (75 g, 0.28 mol) was added portion wise over 5 min and the reaction mixture was stirred for an additional 30 to 60 min. The reaction mixture was cooled to room temperature and diluted with hexanes to facilitate precipitation. The solids were filtered, washed with hexanes and acetonitrile and dried under vacuum to afford the desired product (60 g, 94%) as an off white solid: ^1H NMR (500 MHz, DMSO-d_6) δ 12.48 (bs, 1H), 8.45 (d, J = 5.2 Hz, 1H), 8.00 (d, J = 8.9 Hz, 1H), 7.40 – 7.37 (m, 1H), 7.21 (d, J = 8.9 Hz, 1H), 7.01 – 6.99 (m, 1H), 3.96 (s, 3H), 2.61 (s, 3H); ESI MS *m/z* 219 $[M + H]^+$.

1-(4,6-Dihydroxy-1,5-naphthyridin-3-yl)ethanone. To a suspension of 1-(4-hydroxy-6-methoxy-1,5-naphthyridin-3-yl)ethanone (70 g, 0.32 mol) in acetonitrile (800 ml) was added trimethylsilylchloride (170 g, 1.6 mol) and sodium iodide (140 g, 0.96 mol) and the reaction mixture was heated at reflux for 2 hours. The reaction mixture was cooled to room temperature and satd. aq. sodium thiosulfate was added (100 mL). The mixture was concentrated to remove acetonitrile, diluted with brine (200 mL) and the solids were filtered and dried to provide 1-(4,6-dihydroxy-1,5-naphthyridin-3-yl)ethanone which was carried forward without further purification.

1-(4,6-Dichloro-1,5-naphthyridin-3-yl)ethanone. To a suspension of crude 1-(4,6-dihydroxy-1,5-naphthyridin-3-yl) ethanone in dichloroethane (350 mL) was added phosphorus oxychloride (200 mL) and catalytic *N*,*N*-dimethylformamide and the reaction mixture was heated at 70 °C for 3 hours. The reaction mixture was cooled to room temperature and quenched by pouring slowly into ice cold satd. aq. sodium bicarbonate (1 L). The quenched reaction mixture was concentrated to remove the dichloroethane and the resulting solids were filtered and purified by chromatography (silica, hexanes/ethyl acetate) to provide the desired product (50 g, 74% over 2 steps, **6**) as a brown solid: ^1H NMR (300 MHz, $CDCl_3$) δ 9.00 (s, 1H), 8.38 (d, J = 8.8 Hz, 1H), 7.74 (d, J = 8.8 Hz, 1H), 4.30 (q, J = 7.1 Hz, 2H), 3.28 – 3.18 (m, 1H), 1.36 (t, J = 7.1 Hz, 3H), ESI MS *m/z* 241 $[M + H]^+$.

1-(6-Chloro-4-(*trans*-4-((dimethylamino)methyl)cyclohexylamino) -1,5-naphthyridin-3-yl)-ethanone. To a suspension of 1-(4,6-dichloro-1,5-naphthyridin-3-yl)ethanone (14 g, 59 mmol) in dioxane (250 mL) was added *trans*-4-((dimethylamino)methyl)cyclohexanamine (10 g, 65 mmol) and *N*,*N*-diisopropylethylamine (31 mL, 180 mmol) and the reaction mixture was heated at 80 °C until 1-(4,6-dichloro-1,5-naphthyridin-3-yl)ethanone was consumed (monitored by LCMS analysis). The reaction mixture was cooled, diluted with satd. aq. sodium bicarbonate (500 ml) and extracted with ethyl acetate (3 × 150 mL). The combined organic layers were dried over anhydrous sodium sulfate, filtered and the filtrate was concentrated. The residue was purified by column chromatography (silica, methanol/dichloromethane) to afford the desired product (13 g, 60%) as a yellow solid: ^1H NMR (500 MHz, $CDCl_3$) δ 10.89 (s, 1H), 8.93 (s, 1H), 8.07 (d, *J* = 8.6 Hz, 1H), 7.51 (d, *J* = 8.6 Hz, 1H), 5.16 – 4.96 (m, 1H), 2.67 (s, 3H), 2.34 – 2.24 (m, 2H), 2.22 (s, 6H), 2.14 (d, *J* = 7.1 Hz, 2H), 1.98 – 1.89 (m, 2H), 1.56 – 1.47

(m, 1H), 1.41 – 1.32 (m, 2H), 1.28 – 1.10 (m, 2H); ESI MS m/z 361 $[M + H]^+$; HPLC 98.8% (AUC), t_R = 8.42 min.

1-(6-(3,5-Dichloro-4-hydroxyphenyl)-4-((*trans*-4-((dimethylamino)methyl) cyclohexyl)amino)-1,5-naphthyridin-3-yl)ethanone. To a suspension of 1-(6-chloro-4-(*trans*-4-((dimethylamino)methyl)cyclohexylamino)-1,5-naphthyridin- 3-yl)ethanone (8.6 g, 24 mmol), 2,6-dichloro-4-(4,4,5,5-tetramethyl- 1,3,2-dioxaborolan-2-yl)phenol (8.2 g, 29 mmol) and Pd(dppf)Cl_2 (870 mg, 1.2 mmol) in dioxane (120 mL) was added 1 M Cs_2CO_3 (60 mL, 60 mmol). The reaction mixture was degassed with nitrogen for 10 min and heated at 75 °C for 2 hours. The reaction mixture was cooled and slowly poured into a mixture of ethyl acetate / water / satd. aq. $NaHCO_3$ solution 6:1:1 (400 mL). The mixture was allowed to stir at room temperature for 1 hour then the he solids were filtered and washed with water and acetonitrile. The brown-yellow solid was purified by chromatography (silica, methanol / methylene chloride gradient) to afford the desired product (9.8 g, 85%) as a yellow solid: ^1H NMR (500 MHz, CD_3OD + TFA-d) δ 9.15 (s, 1H), 8.45 (d, J = 9.0 Hz, 1H), 8.34 (d, J = 9.0 Hz, 1H), 8.10 (s, 2H), 5.72 – 5.67 (m, 1H), 3.09 (d, J = 6.5 Hz, 2H), 2.94 (s, 6H), 2.76 (s, 3H), 2.48 – 2.41 (m, 2H), 2.09 – 1.98 (m, 3H), 1.75 – 1.63 (m, 2H), 1.48 – 1.36 (m, 2H). ESI MS *m/z* 487 $[M + H]^+$; HPLC >99% (AUC), t_R = 9.89 min. Elemental analysis for $C_{25}H_{28}Cl_2N_4O_2$: cal'd C, 61.60; H, 5.79; N, 11.49 found C, 61.28; H, 5.77; N, 11.40.

1-(6-(3,5-Dichloro-4-hydroxyphenyl)-4-((*trans*-4-((dimethylamino)methyl) cyclohexyl)amino)-1,5-naphthyridin-3-yl)))ethanone dihydrochloride. To a suspension of 1-(6-(3,5-dichloro-4-hydroxyphenyl)-4-((4-((dimethylamino) methyl)cyclohexyl)amino)-1,5-naphthyridin-3-yl)ethanone (32 g, 66 mmol) in methanol (1 L) was added 4 M HCl in 1,4-dioxane (66 mL) at room temperature. The reaction mixture was stirred until a thick suspension formed and then methanol (2 L) and dichloromethane (500 mL) were added to the reaction mixture to generate a clear solution which was stirred for 16 hours at room temperature. The reaction mixture was concentrated, re-dissolved in dichloromethane (1.5 L) and methanol (2 L) followed by the addition of PL-TMT-MP palladium scavenging resin (2.5 g). The reaction mixture was stirred for 16 h at room temperature, filtered and concentrated to provide a yellow solid. The solid was triturated in a 1:1 mixture of MTBE and dichloromethane (800 mL), filtered, washed with hexanes (3 × 100 mL) and dried under vacuum to obtain the final product (35 g, 95%) as a light yellow solid: ^1H NMR (500 MHz, CD_3OD) δ 9.15 (s, 1H), 8.46 (d, J = 9.0 Hz, 1H), 8.34 (d, J = 9.0 Hz, 1H), 8.11 (s, 2H), 5.72 – 5.67 (m, 1H), 3.09 (d, J = 6.5 Hz, 2H), 2.94 (s, 6H), 2.76 (s, 3H), 2.48 – 2.41 (m, 2H), 2.09 – 1.98 (m, 3H), 1.75 – 1.63 (m, 2H), 1.48 – 1.36 (m, 2H). ESI MS *m/z* 487 $[M + H]^+$; HPLC >99% (AUC), t_R = 9.89 min. Elemental analysis for $C_{25}H_{28}Cl_2N_4O_2$ • 2HCl • 0.5 H_2O: cal'd C, 52.74; H, 5.49; N, 9.84 found C, 52.50; H, 5.41; N, 9.73.

Synthesis of *trans*-4-((dimethylamino)methyl)cyclohexanamine

***tert*-Butyl *trans*-4-((dimethylamino)methyl)cyclohexylcarbamate.** To a solution of *tert*-butyl *trans*-4-(aminomethyl)cyclohexylcarbamate (1.0 g, 4.5 mmol), paraformaldehyde (410 mg, 13 mmol), and sodium cyanoborohydride (840 mg, 13mmol) in methanol (40 mL) was added acetic acid (catalytic) and the reaction mixture stirred at room temperature for 18 hours. The reaction mixture was quenched

with water and concentrated to remove methanol. The pH of the aqueous layer was adjusted to 10 with 1 M aqueous sodium hydroxide followed by extraction with methylene chloride. The organic layer was dried over anhydrous sodium sulfate, filtered, and concentrated to afford the desired product (1.1 g, 96%) as an oil: ESI MS *m/z* 257 [$C_{14}H_{28}N_2O_2$ + H]$^+$.

***trans*-4-((dimethylamino)methyl)cyclohexanamine.** To a suspension of *tert*-butyl *trans*-4-((dimethylamino)methyl)cyclohexylcarbamate (1.1 g, 4.30 mmol) in THF (0.1 M) was added 3 M hydrochloric acid (10 mL) and the reaction mixture was heated at 65 °C until the reaction was complete, as observed by LCMS analysis. The reaction mixture was cooled and concentrated to obtain the desired product (1.0 g, >99%) as a glass: ESI MS *m/z* 230 [$C_9H_{20}N_2$ + H]$^+$.

Synthesis of 2,6-Dichloro-4-(4,4,5,5-tetramethyl-1,3,2-dioxaborolan-2-yl)phenol. To a suspension of 4-bromo-2,6-dichlorophenol (290 mg, 1.20 mmol) bis(pinacolado)diboron (300 mg, 1.2 mmol) and Cs_2CO_3 (1.2 g, 3.6 mmol)) in dioxane (15 mL) was added Pd(dppf)Cl_2 (85 mg, 0.12 mmol)). The reaction mixture was degassed with nitrogen for 5 min followed by heating at 80 °C for 16 hours. The reaction mixture was cooled, concentrated and the residue was purified by column chromatography (silica, ethyl acetate/hexanes gradient) to afford the desired product (298 mg, 86%): ESI MS *m/z* 289 [$C_{12}H_{15}BCl_2O_3$ + H]$^+$.

Supplementary Figure

Fig S1. Effect of OTSSP167 on body weight for mice xenograft models.

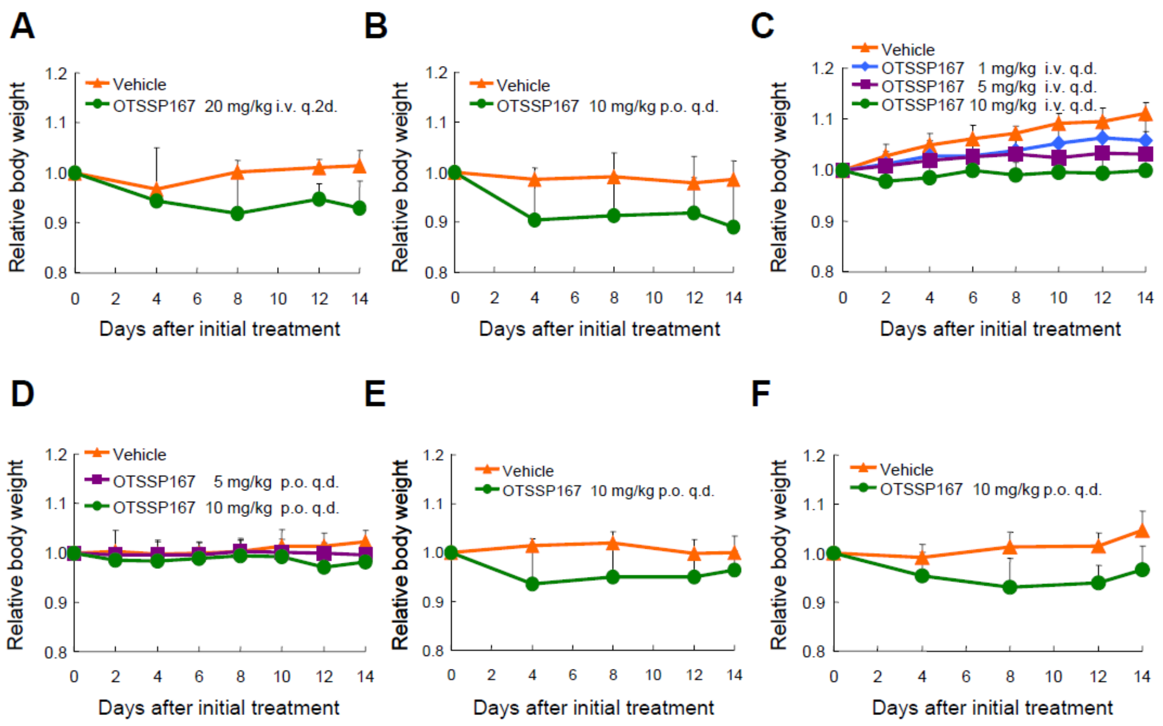

Nude mice bearing (**A,B**) MDA-MB-231 (MELK-positive, triple negative breast cancer), (**C,D**) A549 (lung cancer), (**E**) DU145 (prostate cancer), or (**F**) MIAPaCa-2 (pancreatic cancer) cells were administered either vehicle control or OTSSP167 for 14 days. Mean relative body weights ± SD (n = 6 per each treatment group) in comparison with the mean body weight just before the administration (day 0) are shown. The mean relative body weights after 14 days of administration were: (**A**) 0.93 for 20 mg/kg intravenously once every two days. in MDA-MB-231; (**B**) 0.89 for 10 mg/kg orally once a day in MDA-MB-231; (**C**) 1.06 for 1 mg/kg intravenously once a day, 1.03 for 5 mg/kg intravenously once a day, and 1.00 for 10 mg/kg intravenously once a day in A549; (**D**) 0.99 for 5 mg/kg orally once a day, and 0.98 for 10 mg/kg orally once a day in A549; (**E**) 0.96 for 10 mg/kg orally once a day in DU145; (**F**) 0.97 for 10 mg/kg orally once a day in MIAPaCa-2. i.v. q.2d; intravenously once every two days, i.v. q.d.; intravenously once a day, p.o. q.d.; orally once a day.
